# Osteolipoma of head and neck – a review

**DOI:** 10.1016/j.bjorl.2022.04.002

**Published:** 2022-05-20

**Authors:** Billy L.K. Wong, Christopher Hogan

**Affiliations:** Mid and South Essex NHS Foundation Trust, Broomfield Hospital, Department of Otolaryngology, Chelmsford, Essex, CM1 7ET, United Kingdom

**Keywords:** Osteolipoma, Head and neck, Radiology, Histopathology

## Abstract

**Objective:**

Due to the rarity of osteolipoma, current knowledge and understanding of its’ clinical presentation, management, radiological features, histological characteristics, and prognosis are lacking and can present a clinical conundrum to clinicians and histopathologist alike, given wide range of differential diagnoses. This paper aims to compile, analyse and present details to augment the available literature on osteolipoma in the head and neck.

**Methods:**

A comprehensive literature search on PUBMED/MEDLINE, EMBASE, CINAHL and Science Citation Index, Google scholar and Cochrane database for osteolipoma in head and neck was performed up to the 1st May 2021. Reference lists from the relevant articles were then inspected and cross-referenced and any other pertinent publications were added to the review.

**Results:**

A total of 38 cases were identified from the literature. The commonest sites of involvement within the head and neck region were the oral cavity in 21 (56.8%) patients, followed by the neck in 7 (19.0%) patients. 29 (78.4%) patients presented with soft tissue swelling or mass making it the most common presenting feature. All patients had the tumour excised surgically; of which 18 (48.6%) were excised via transoral approach and 6 (16.2%) via open transcervical approach including 1 lateral pharyngotomy. 12 papers documented long-term follow-ups (median 12 months) with no recurrence. Only 1 regrowth was reported after 5-years.

**Conclusions:**

Osteolipoma is a rare soft tissue neoplasm which has a wide range differential diagnosis including malignant processes. Recognising this benign tumour through an awareness of presenting sign and symptoms, radiological features and histopathology findings is important for patient reassurance as well as avoiding unnecessary radical treatment.

## Highlights

• 80% of the patients with osteolipoma presented with a painless mass which had pre-existed for at least 4-months contrary to a lump associated with a malignant process.
• Histology consisting of variable mixture of adipose tissue interspersed with lamellar bone, woven bone, cancellous bone, compact bone and osteoblasts is key to confirm the diagnosis of osteolipoma.
• Osteolipoma should not be confused with other benign tumours with bony element including parosteal lipomas and intraosseous lipomas.
• Recognising osteolipoma early is important for patient reassurance as well as avoiding unnecessary radical treatment.

## Introduction

Osteolipoma was first described by Plaut et al. in 1959.^1^ Over the years, it has been given different names including osseous lipoma, ossifying lipoma and lipoma with osseous metaplasia.^2^

According to the World Health Organisation (WHO) classification of soft tissue tumours, osteolipoma is a variant of lipoma. It is an extremely rare variant, accounting for less than one percent of all lipomas found in the human body.^3^, ^4^

It is even rarer within the head and neck region, with only 37 cases described in the English literature. Owing to the rarity, comprehensive details about its clinical presentation, management, radiological features, histological characteristics and prognosis are lacking and presents a clinical conundrum to clinicians.

In this review, we aim to examine the cases in the literature and present a complete updated review on this rare tumour.

## Methods

A comprehensive literature search on PUBMED/MEDLINE, EMBASE, CINAHL and Science Citation Index, Google scholar and Cochrane database for osteolipoma in head and neck was performed up to the 1st May 2021. Key words used were osteolipoma, ossifying lipoma, ossified lipoma, lipoma with osseous metaplasia and head and neck. The search was further extended MeSH words like oropharynx; tonsil; tongue base; palate; larynx; pharynx; hypopharynx; pyriform fossa; postcricoid region; lateral pharyngeal wall; neck; parotid; submandibular; oral cavity; external auditory canal; internal auditory canal; temporal bone; paranasal sinus; tongue; and mandible. All articles including non-English papers were considered, and if included, non-English papers were translated.

**Figure 1:**
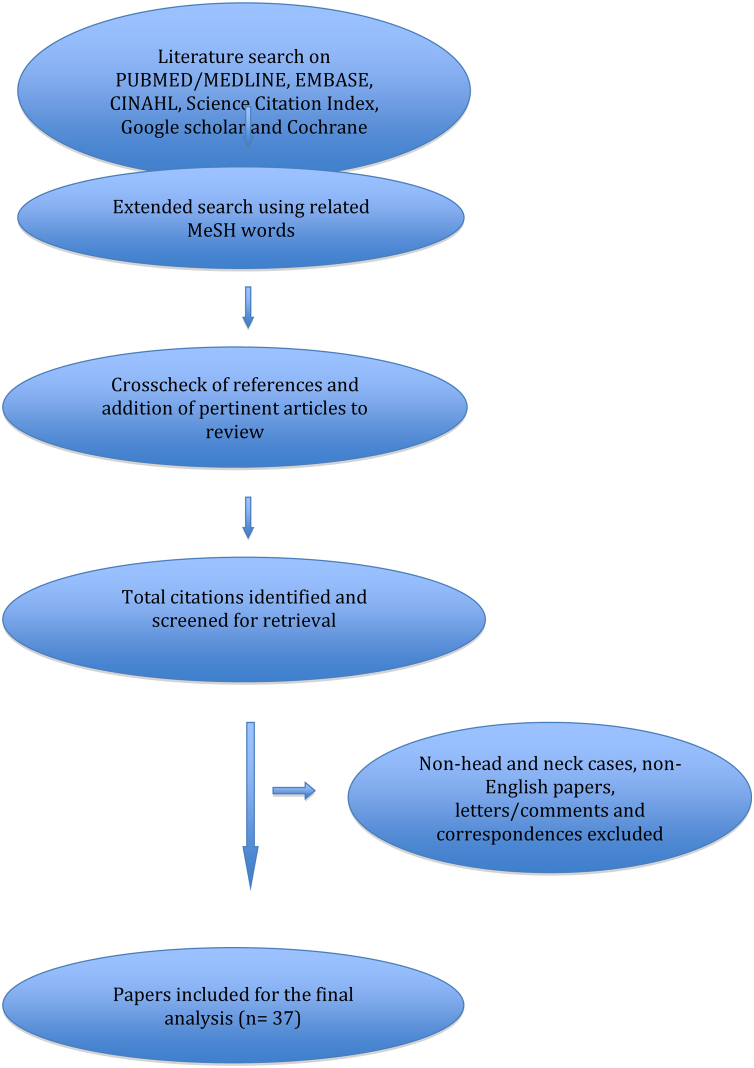
PRISMA flow diagram of the literature search.

Publications on osteolipoma arising only from the head and neck sub-sites were included. Reference lists from the relevant articles were then inspected and cross-referenced and any other pertinent publications were added to the review. Detailed search strategy is shown in Fig. 1.

## Results

A total of 38 cases were identified and included from the literature (Table 1). All the papers were in English, except one (Turkish). There were 19 (50%) females and 19 (50%) males. The patients’ age ranged from 6-years to 81-years, with a median age of 51. Three quarters of the patients were between 30 and 70-years of age. Only one paediatric case was ever reported.

**Table 1:** Cases from the literature.

Author	Site	Subsite	Gender	Age	Duration	Presentation	Imaging	Size	Treatment	Outcome
Abdalla^5^	Paranasal sinus	Frontoethmoidal sinus	M	66	ND	Nasal obstruction	CT	ND	Endoscopic anterior ethmoidectomy and partial excision left middle meatus bony mass	Regrowth ‒ further debulking
Aboh^6^	Neck	Submandibular area	M	33	ND	Painless mass	MRI, CT	ND	Transcervical excision	ND
Adebiyi^7^	Oral cavity	Hard palate	F	37	10 years	Painless mass	Occlusal radiograph	3.2 × 2 × 1.6 cm	Transoral excision	ND
Allard^8^	Oral cavity	Mandibular buccal sulcus	F	81	30 years	Painless mass	Radiograph	3.5 × 2 cm	Transoral excision	ND
Amaral^9^	Oral cavity	Buccal mucosa	M	51	3 years	Painless mass	US, radiography	2.0 × 1.5 cm	Transoral excision	No recurrence after 12 months
Arantes^10^	Neck	Mandibular area	F	60	5 years	Painless mass	OPG, CT	2.6 × 1.6 × 0.9 cm	Transoral excision	No recurrence after 12 months
Bajpai^11^	Oral cavity	Hard palate	M	55	4 years	Painless mass	Occlusal radiograph	1.5 × 1 cm	Transoral excision	ND
Battaglia^12^	Salivary gland	Parotid gland	M	56	15 years	Painless mass	CT, MRI	4.3 × 3.7 × 6 cm	Parotidectomy	ND
Blanshard^13^	Pharynx	Retropharynx	M	40	4 months	Painless mass	Radiograph, CT	3 cm	Lateral pharyngotomy approach	ND
Bowers^14^	Pharynx	Retropharynx	M	81	2 years	Dysphagia, weight loss	MRI, CT	2 × 4 × 6 cm	Transcervical excision	ND
Bulkeley^15^	Neck	Parapharyngeal space	M	68	ND	Jaw pain, numbness in V1 distribution		4 × 1.5 × 1 cm	Transcervical excision	ND
Castilho^16^	Oral cavity	Buccal mucosa	F	65	ND	Painless mass	NA	1 × 1 × 0.8 cm	Transoral excision	ND
Decastro^17^	Oral cavity	Buccal mucosa	F	47	ND	Facial mass	NA	1.5 cm	Excision of lesion	ND
Diom^18^	Salivary gland	Parotid gland	F	21	1 year	Painless mass	NA	5 cm	Parotidectomy	No recurrence after 26 months
Dougherty^2^	Oral cavity	Gingivolabial sulcus	F	30	6 years	Facial mass	CT	4 × 2.5 cm	Transoral excision	ND
Durmaz^19^	Nasopharynx	Nasopharynx	M	21	5 years	Aural fullness, nasal obstruction	CT, MRI	3 × 2 cm	Transnasal endoscopic and transpalatal approach	No recurrence after 6 months
Firth^20^	Oral cavity	Buccal mucosa	F	56	ND	Painless mass	CT	1.8 × 1.2 × 0.8 cm	Excision of lesion	ND
Fukushima^21^	Face	Zygomatic arch/coronoid process	M	28	13 years	Trismus, facial mass	CT, MRI	6.6 × 4.5 × 2.1 cm	Intra and extra oral excision	No recurrence after 24 months
Godby^22^	Oral cavity	Floor of mouth	M	54	1 year	Painless mass	Radiograph	7 × 6 × 3 cm	Transoral excision	ND
Gokul^23^	Oral cavity	Hard palate	M	6	6 years	Nasal regurgitation, recurrent OME	CT	3 × 2 cm	Transoral excision	ND
Hazarika^24^	Neck	Parapharyngeal space	F	17	1 year	Facial mass, lump in throat	CT	5 × 4 cm	Mandibulotomy and transcervical excision	No recurrence after 1 month
Hsu^25^	Oral cavity	Buccal mucosa	M	71	4 years	Painless mass	NA	3.8 × 2.4 × 1.3 cm	Excision of lesion	No recurrence after 12 months
Hughes^26^	Oral cavity	Mandibular buccal vestibule	M	69	ND	Painless mass	OPG	3.5 × 2.6 × 1.7 cm	Transoral excision	ND
Cakir Karabas^27^	Oral cavity	Mandibular buccal vestibule	M	53	ND	Painless mass	Radiograph, CBCT	2 × 15 × 1 cm	Excision of lesion	ND
Kavusi^28^	Neck	Submandibular area	M	67	10 years	Painless mass	CT	3.5 × 3 × 2 cm	Transcervical excision	ND
Kumar^29^	Eye	Eyelid	F	50	5 years	Nodular swelling	NA	2 × 1.5 × 1 cm	Excision of lesion	ND
Minutoli^30^	Neck	Parapharyngeal space	F	46	ND	Dysphagia, paraesthesia V3, OME	CT, MRI	2.5 × 4 cm	Transcervical excision	ND
Ohno^31^	Neck	Parapharyngeal space	F	58	1 year	Throat and neck mass	CT, MRI, gallium, 99mTc	9 × 4 cm	Transcervical and transoral approach	ND
Omonte^32^	Oral cavity	Buccal mucosa	F	29	8 months	Painless mass	Radiograph	1.8 × 1.5 × 1.2 cm	Transoral excision	No recurrence after 5 years
Piattelli^33^	Oral cavity	Tongue	F	49	8 years	Painless mass	NA	0.8 cm	Transoral excision	No recurrence after 4 years
Raghunath^34^	Oral cavity	Floor of mouth	F	20	3 years	Painless mass	CT	6 × 6 cm	Transoral excision	ND
Ramadass^35^	Mastoid	External auditory meatus/mastoid	M	45	18 years	Painless mass	CT	3 × 4 cm	Excision of lesion ‒ post auricular approach	ND
Raviraj^36^	Oral cavity	Buccal mucosa	F	38	10 years	Painless mass	OPG	2 × 2 × 3 cm	Transoral excision	ND
Saghafi^37^	Oral cavity	Mandibular Alveolar mucosa	M	68	4 years	Painless mass	Radiograph	1.5 × 1 cm	Transoral excision	No recurrence after 12 months
Seelam^38^	Oral cavity	Retromolar region	F	55	6 years	Painless mass	US, OPG	3 × 2 cm	Transoral excision	ND
Shabbir^39^	Oral cavity	Labial sulcus	F	58	1 year	Painless mass	OPG	2 × 2 cm	Transoral excision	ND
Sharma^40^	Oral cavity	Hard palate	M	35	8 years	Painless mass	CT	4 × 2.7 × 0.8 cm	Transoral excision	No recurrence after 36 months
Turkoz^48^	Mastoid	Mastoid	F	34	6 years	Painless mass	US	3 × 2.5 × 1.5	Excision of lesion	No recurrence after 12 months

F, Female; M, Male; CT, Computed Tomography; CBCT, Cone Beam CT; MRI, Magnetic Resonance Imaging; US, Ultrasound; OPG, Orthopantomogram; ND, Not Described.

The commonest sites of involvement within the head and neck region was the oral cavity in 21 (56.8%) patients, followed by the neck in 7 (19.0%) patients. Other reported sites included the salivary gland, paranasal sinuses, nasopharynx, pharynx, orbit, and tympanomastoid region. The smallest recorded lesion was 8-mm in size within the tongue and the largest lesion measured 7 × 6 cm on the floor of mouth.

30 (78.4%) patients presented with soft tissue swelling or mass making it the most common presenting feature. Other common presenting features include dysphagia (5.4%), paraesthesia in the trigeminal distribution (5.4%), nasal obstruction (5.4%) and middle ear effusion (5.4%).

All patients had the tumour excised surgically; of which 18 (48.6%) were excised via transoral approach and 6 (16.2%) via open transcervical approach including 1 lateral pharyngotomy. Other patients required combined transoral and transcervical approaches (5.4%), parotidectomies (5.4%) and transnasal endoscopic approach (5.4%) respectively.

13 papers documented follow-up ranging from 1-month to 5-years (median 12-months) with no recurrence. Only 1 regrowth was reported after 5-years.

## Discussion

### Terminology

The terminologies used for adipocytic tumour/lipoma with osseous component can be confusing. Terms such as ossifying lipoma, osseous lipoma, and lipoma with osseous metaplasia have been used interchangeably with osteolipoma. Consequently, many prior reports of “osteolipoma” included tumours which in actual fact are parosteal lipomas and intraosseous lipomas.^28^, ^31^, ^32^, ^41^

Parosteal lipomas are neoplasms of mature adipose tissues that are contiguous with underlying periosteal bones, commonly associated with reactive changes or hyperostosis in the underlying cortex whereas intraosseous lipomas are lipomas that arises within the medullary cavity and occasionally within the cortex of a bone.^2^, ^28^, ^31^, ^32^^,^^41^

Hence, “true” osteolipomas, based on the current review are adipocytic neoplasms with osseous tissue which are independent or not attached to any bone.

Osteolipoma however, can be classified according to the composition of its tissue content. It is called ossifying lipoma if the adipose component is the predominant tissue type, while the term osteolipoma is used if it contains more bony element.^42^

### Epidemiology

Osteolipoma is rare and only featured in case series and case reports to date. Its’ precise incidence is therefore unknown. However, it is thought to account for less than 1% of all lipoma cases.^28^, ^43^

It has near-equal gender ratio and is pre-dominantly found in adulthood. Gokul et al. reported a congenital case in a patient aged 6, of the hard palate. It remains the only paediatric case reported in the literature to date.^23^

### Presentation

**Figure 2:**
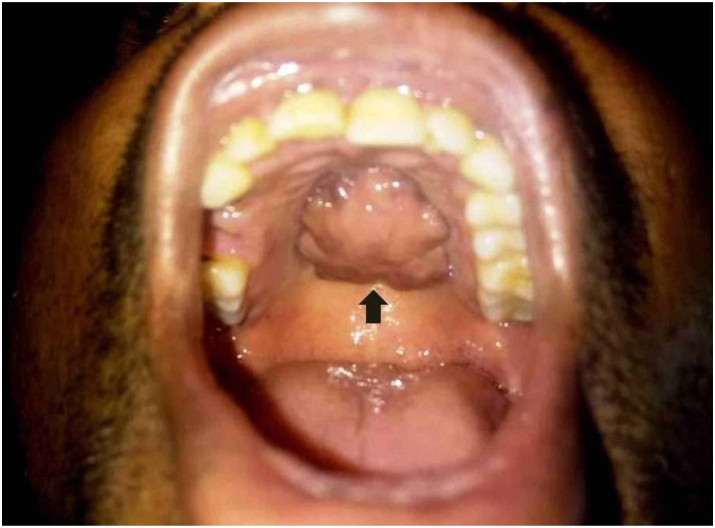
A clinical photograph on an osteolipoma of the hard palate in the oral cavity.

Classically, osteolipoma and majority of the head and neck osteolipoma presents as a painless swelling or mass located in the submucosa or soft tissue (Fig. 2). The texture or consistency of the mass itself is variable ranging from soft and fluctuant to firm and hard.^33^, ^38^ The surface of the mass can be smooth or nodular with no overlying skin or mucosal changes.^2^

Additionally, when arising from the head and neck region, the signs and symptoms can be variable depending on the site of origin and size of the tumor. Lesions arising from the nasopharynx and paranasal sinuses tend to present with nasal obstruction.^5^, ^19^ Large tumor located in pharynx or parapharyngeal space can present with pressure symptoms on the surrounding structures, resulting in dysphagia, paraesthesia in the trigeminal nerve distribution and middle ear effusion.^14^, ^15^, ^30^

The child with the congenital osteolipoma of the palate presented with a cleft palate, nasal regurgitation and recurrent middle ear infections.^23^

### Diagnostic imaging

Given the wide range differential diagnoses of soft tissue tumours, characteristic radiological features are vital in aiding the diagnosis of osteolipoma, as well as assessing the exact site, delineate the extent of disease and help decide treatment approaches.

**Figure 3:**
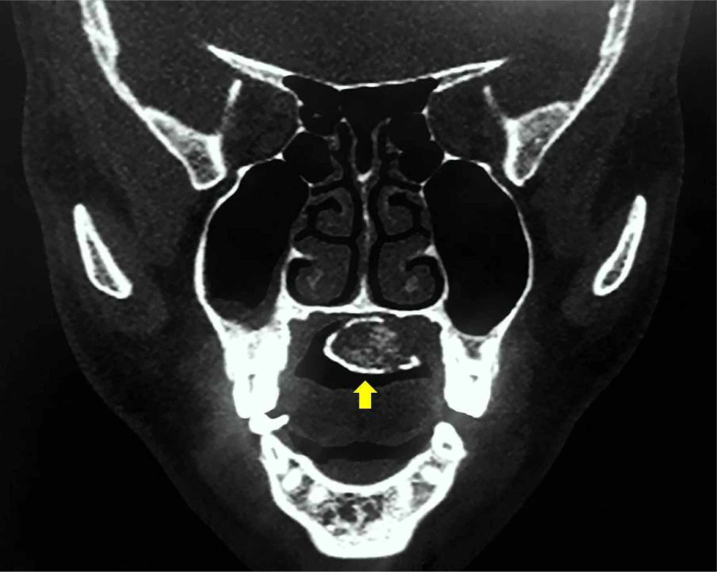
A coronal CT scan of the patient in Fig. 2 showing a hypodense mass (fat attenuation centrally) with peripheral hyperattenuation (calcification).

Computed Tomography (CT) with or without contrast is the most frequently used cross-sectional imaging to investigate osteolipoma. Radiologically, it is a well-defined, heterogenous mass with mixed density.^2^, ^18^ Its overall appearance is dependent on the predominant make-up of the tumour. Tumours which are clinically soft and consist of mainly fatty tissue appear as hypodense mass (fat attenuation centrally) with peripheral hyperattenuation (calcification) (Fig. 3).^24^, ^40^ Internal septations can occasionally be observed.^15^, ^40^ Conversely, tumours which are clinically firm and hard would appear as a hyperdense mass with central calcified portion and focal areas of fat attenuation.^2^, ^10^

More importantly, osteolipoma does not erode into the underlying bone, nor does it invade into the surrounding structures which would suggest a more aggressive and sinister disease process.^2^, ^40^ It is also not known to cause periosteal reaction or hyperostosis of the adjacent bone. However, it can displace adjacent structures and cause thinning and bowing of the adjacent bones.^15^, ^21^, ^24^, ^30^

**Figure 4:**
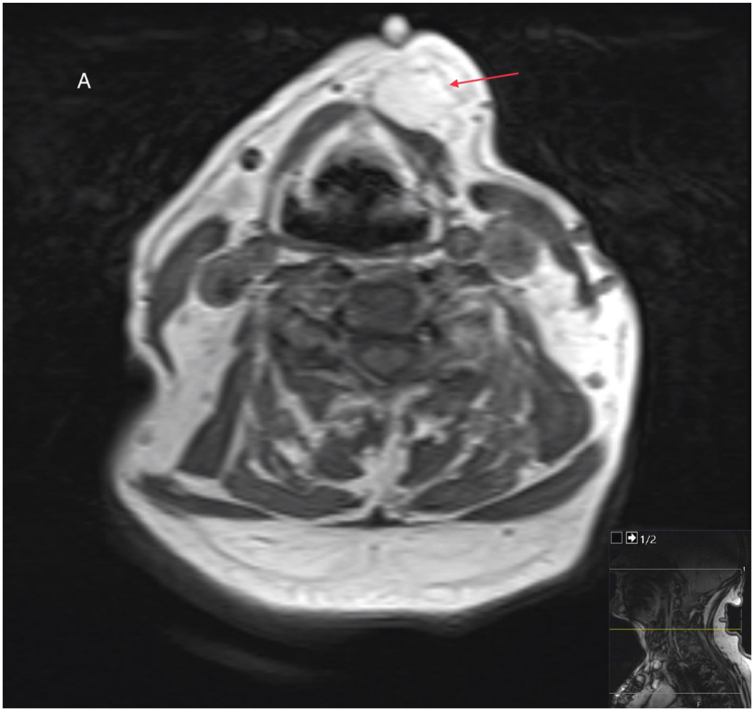
An axial MRI of another patient (histologically confirmed osteolipoma) showing a well circumscribed tumour with high signal intensity on T1.

Although Magnetic Resonance Imaging (MRI) is the imaging modality of choice to characterize soft tissue neoplasm, it was only utilized in 7-cases, most likely due to limited availability. On MRI, osteolipoma is a well circumscribed tumour with high signal intensity on T1 (Fig. 4) and Short Tau Inversion Recovery (STIR) sequences. It has a suppressed signal intensity on Diffusion-Weighted magnetic resonance Imaging (DWI) sequences and fat-suppressed images. Hence, it is important to obtain images with fat suppression to differentiate fatty tissue from other soft tissues on magnetic resonance imaging.^12^, ^18^, ^19^ It can, however, appear either as a hyperintense or hypointense lesion on T2-weighted images depending on its’ core component. The lining of the tumour is often hypointense on both T1 and T2 images due to the osseous layer or fibrous tissue circumscribing the lesion.^12^, ^19^, ^30^, ^40^

Occlusal radiograph or orthopantomography is useful as first line investigation for tumour arising within the oral cavity. Typically, oesteolipoma would appear as a well-defined radiopaque mass with an irregular pattern of trabeculae or occasional flecks of calcification within. Given the nature of soft tissue tumour, no cortical abnormality should be found.^8^, ^32^, ^39^

Ultrasonography was only used in a handful of cases. It would characterize the lesion as hyperechoic with focal areas of calcification.^9^, ^38^

### Histopathology

Histologically, osteolipoma consists of mature adipose tissue interspersed with multi-focal areas of bony tissue.^24^, ^29^ It contains a variable mixture of adipose tissue, lamellar bone, woven bone, cancellous bone, compact bone and osteoblasts.^5^ The lobules of adipose tissue are separated by thin fibrous connective tissue septa.^10^, ^17^, ^32^

Microscopically, the adipocytes are regular in size and shape, and the nuclei are uniform with no hyperchromasia.^12^ The bony tissue can be mature or immature. The more mature lamellar bone exhibits Haversian canal formation and central fatty marrow which lacks hematopoietic cells.^14^, ^32^ On the other hand, irregular trabeculae of woven bone are surrounded by proliferating osteoblasts and active collagenisation.^24^, ^29^ Some of the mature bony tissues were also lined with osteoblasts.^21^

No nuclear atypia, cellular pleomorphism, mitosis or necrosis was ever reported in any of the cases.

### Pathogenesis

The pathogenesis of osteolipoma is still unclear. Several hypotheses have been proposed to explain its’ pathogenesis.

It is thought that osteolipomas may arise from the proliferation of mesenchymal stem cells (characterizing a “mesenchymoma”), either directly from multipotent stem cells, or cells from a different lineage which subsequently differentiates into lipoblasts, chondroblasts or osteoblasts, and fibroblasts.^8^, ^17^, ^20^, ^27^ This adipo-osteogenic differentiation of mesenchymal stem cells is finely balanced by a variety of external factors including chemical, physical, and biological factors.^44^

Another hypothesis is that osteolipoma forms from fibroblast metaplasia within a pre-existing lipoma, which is usually large and long-standing.^16^, ^31^ The metaplasia occurs due to repetitive trauma which leads to subsequent metabolic changes, ischemia and calcium deposition.^2^, ^37^ Fritchie et al. was in support of this theory as cytogenetic analyses from their case series showed chromosomal translocations which are consistent with the karyotypic features of simple lipoma.^43^ Arantes et al. also indicated that the presence of osseous of trabeculae along the fibrous septi further supported this hypothesis.^10^

Other alternative hypothesis includes the transformation of fibroblasts into osteoblasts induced by growth factors released from monocytes or due to the ossification of an inadequately nutritional supplied tissue within the core the lipoma.^13^, ^27^, ^31^

### Differentials

The differential diagnoses for a well-defined, extraosseous soft-tissue mass containing both adipose and osseous components is dependent on the location of the lesion. Nonetheless, the differential diagnoses are wide and includes both benign and malignant processes as outlined in Table 2.

**Table 2:** Differential diagnoses.

Site	Differential diagnoses
Nasopharynx	Chondroblastoma
	Osteochondroma
	Calcified lipoma
	Ossifying fibroma
	Osteoma
	Enchondroma
Paranasal sinus	Inverted papilloma
	Fibrous dysplasia
Neck	Submental triangle:
	Teratoma
	Tumour calcinosis
	Ossifying fibroma
	Hemangioma
	Myositis ossificans
	Soft tissue sarcomas (liposarcoma, synovial sarcoma, osteosarcoma, chondrosarcoma)
	Parapharyngeal space:
	Calcified lipoma
	Ossifying fibroma
	Osteoma
	Enchondroma
	Teratoma
Oral cavity	Alveolar mucosa:
	Exostosis
	Peripheral giant cell granuloma
	Fibrous hyperplasia
	Fibroma with calcifications
	Buccal mucosa:
	Osteocartilaginous choristoma
	Chrondrolipoma
	Pleomorphic adenoma with ossification
	Mucocele
	Benign minor salivary gland tumour
	Tongue:
	Osteocartilaginous choristoma
	Osteosarcoma
	Liposarcoma with metaplasia
	Post-traumatic chondrofication
	Palate:
	Cementifying fibroma
	Osteoma
	Neurofibroma
	Intraosseous palatal cyst
	Well differentiated liposarcomas
	Floor of mouth:
	Teratoma
	Dermoid cyst
	Osteoma
	Ossifying fibroma
	Myositis ossificans
	Osteocartilaginous choristoma
	Metastatic chondrosarcoma
	Osteosarcoma
	Liposarcoma with metaplasia
Salivary gland (parotid)	Synovial sarcoma

In the paranasal sinuses, the differential diagnosis includes inverted papilloma due to its’ appearance on endoscopy. However, the lack of adjacent inflammatory mucosa on CT is unusual for this entity. Instead, the ground-glass appearance of the lesion on CT suggests that fibrous dysplasia should be considered as a differential diagnosis.^5^

One of the main differential diagnoses of osteolipoma within the oral cavity is osteocartilaginous choristoma. It has a marked predilection for the tongue, but diagnosis can only be confirmed through histopathology. In contrast to osteolipoma, the histology of osteocartilaginous choristoma shows mass of dense lamellar bone with Haversian canals and haematopoietic marrow which is absent in osteolipoma.^27^

Other differential diagnoses within the oral cavity include dermoid cyst, teratoma, myositis ossificans and liposarcoma. Dermoid cyst and teratoma tend to be more heterogenous and cystic on CT. Myositis ossificans has a typical appearance on CT – a well circumscribed intramuscular lesion with a distinct zoning/ossification pattern which progresses from an immature, central, non-ossified cellular focus, to osteoid, and then to a peripheral rim of mature bone over days to weeks. It is separated from the underlying bone by a radiolucent zone. In the head and neck, MO is often found in the pterygoid muscles but can be seen in the masseter, temporalis, buccinators, sternocleidomastoid, and platysma.^2^, ^45^, ^46^

Osteolipoma can mimic or be difficult to differentiate from well-differentiated liposarcoma based on imaging alone. However, features such as thickened or nodular septi (>2 mm thick), prominent foci of high T2 signal and prominent areas of enhancement are suspicious for liposarcoma.^47^

### Management

As a benign, indolent and slow growing neoplasm, osteolipoma can potentially be managed conservatively. Majority of the lesions reported were long-standing, with duration since detection ranging from 4-months to 30-years (median – 4-years). However, most of these patients presented due to the enlarging lesion or symptoms secondary to the compressive or obstructive effect requiring surgical intervention.

The only treatment of choice is therefore a complete surgical excision. The surgical approach differs based on the location of the osteolipoma. 17 of the 21 cases of the oral cavity lesions were excised successfully via a transoral approach. The remaining 4 cases did not describe their approaches.

Parotid salivary gland lesions were managed using a parotidectomy approach. Diom performed a total parotidectomy as the lesion was located between the deep lobe of parotid gland and the parapharyngeal space.^18^ The integrity of the facial nerve was not mentioned. Battaglia performed a superficial parotidectomy as the lesion was entirely within the superficial lobe.^12^ The facial nerve was fully preserved.

Endoscopic approaches were used for lesions within the paranasal sinuses and nasopharynx. Durmaz employed a combination of transnasal and transpalatal approach to excise the lesion attached to the posterior surface of nasal septum and posterior wall of nasopharynx.

Most osteolipoma within the neck were excised using a transcervical approach. One lesion within the parapharyngeal space extending into the infratemporal fossa required a mandibulotomy.^24^ Blanshard performed a lateral pharyngotomy to access an osteolipoma in the retropharynx.^13^

### Prognosis

Osteolipoma has a particularly good prognosis similar to that of a conventional lipoma. Of the 12 cases which documented their long-term follow-ups, no recurrences were reported in the 11 lesions which were completed excised. No malignant transformation had been reported to date. Abdalla et al. reported a regrowth and not recurrence of the lesion 5-years after the initial surgery, where the mass was only partially excised due to the extension into frontal sinus.^5^

There is currently no consensus regarding the duration of follow-up for head and neck osteolipoma. In this review, the longest documented follow up was up to 5-years. Some authors suggested close monitoring and long-term follow-up given the paucity of knowledge about this rare entity.

## Conclusion

Osteolipoma is a rare soft tissue neoplasm which has a wide range differential diagnosis including malignant processes. Recognising this benign tumour through an awareness of presenting sign and symptoms, radiological features and histopathology findings is important for patient reassurance as well as avoiding unnecessary radical treatment.

## Conflicts of interest

The authors declare no have conflicts of interest.
